# Supplementary figures and images for: Effect of Press Cake-Based Particles on Quality and Stability of Plant Oil Emulsions
Source: Foods. 2024 Sep 19;13(18):2969. doi: 10.3390/foods13182969 (PMC11431225; doi:10.3390/foods13182969)

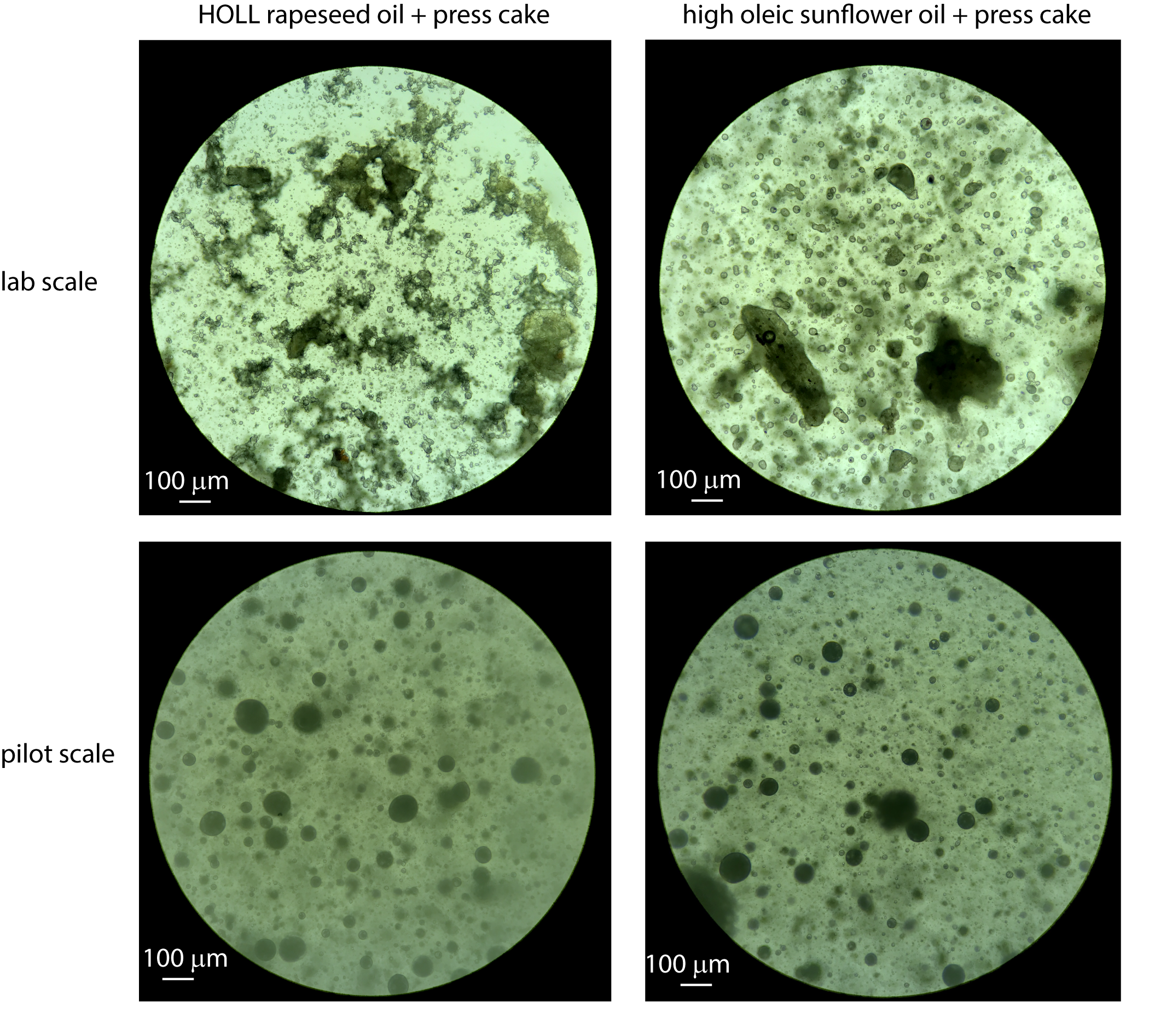

Supplement: Supplementary file 1 [file foods-13-02969-s001.zip › Supp-Fig-S1-Emul-Bilder.tif]

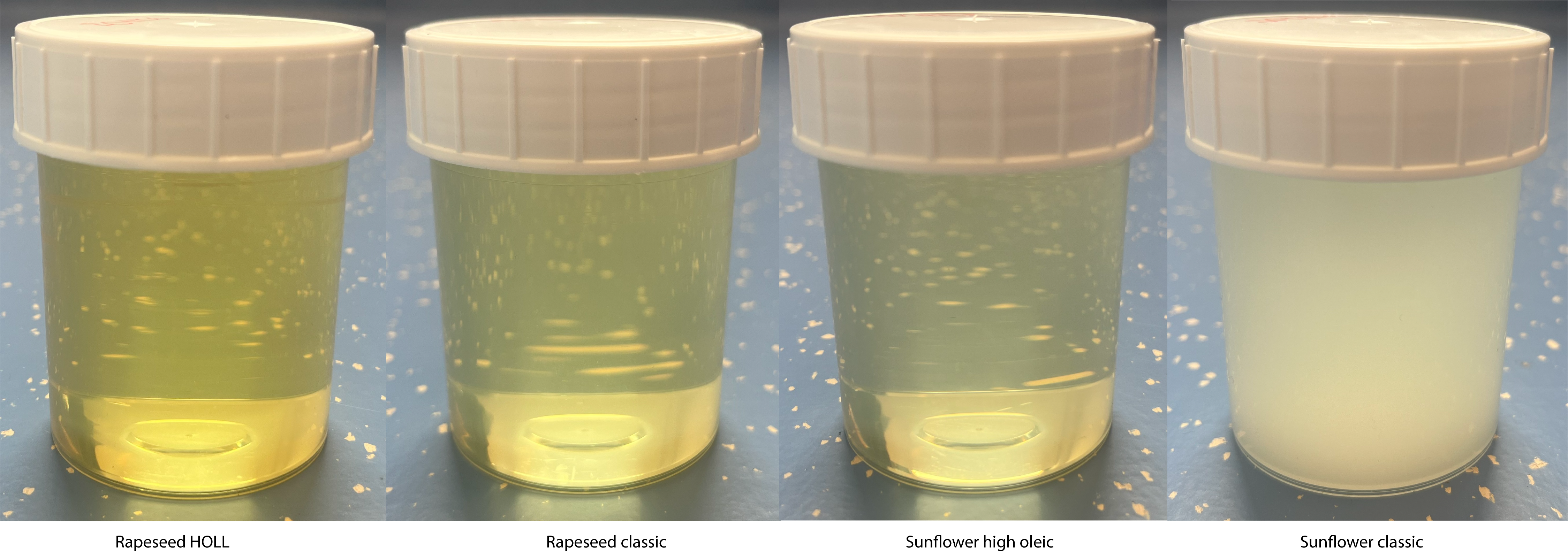

Supplement: Supplementary file 1 [file foods-13-02969-s001.zip › Supp-Fig-S2-Oils.tif]
